# Phase-sensitive small-angle neutron scattering experiment

**DOI:** 10.1088/2399-6528/aadf5f

**Published:** 2018

**Authors:** Erik Brok, Kathryn L Krycka, Erika C Vreeland, Andrew Gomez, Dale L Huber, Charles F Majkrzak

**Affiliations:** 1 NIST Center For Neutron Research, National Institute of Standards and Technology, Gaithersburg, MD 20899, United States of America; 2 Department of Materials Science and Engineering, University of Maryland, College Park, MD 20742, United States of America; 3 IR Dynamics, LLC, Albuquerque, NM 87109, United States of America; 4 Center for Integrated Nanotechnologies, Sandia National Laboratories, Albuquerque, NM 87185, United States of America; 5 Sandia National Laboratories, Albuquerque, NM 87185, United States of America; 6 Now at: Nano Science Center, Niels Bohr Institute, University of Copenhagen, DK-2100 Copenhagen, Denmark

**Keywords:** neutron scattering, biomolecules, core–shell nanoparticle

## Abstract

In the work reported herein, we investigate the practicality of a recently introduced variant of a general phase-sensitive method in small-angle neutron scattering that attempts to address the loss of phase-information as well as the orientational averaging simultaneously—through the use of reference structures in conjunction with finite element analysis. In particular, one possible physical realization of this approach is to employ polarized beams together with a magnetic reference connected to the sample object. We report on a first such practical implementation by successfully recovering the structure of a core–shell nanoparticle system.

## 1. Introduction

In typical diffraction experiments information about the phase of the scattered wave is lost because the measured quantity is the squared modulus of the structure factor and not the structure factor itself. The loss of phase information leads to ambiguity in structure determination from diffraction data. This so-called phase problem is arguably the biggest problem in diffraction experiments. Methods such as isomorphic substitution of atoms in crystals, resonant x-ray scattering at synchrotrons, and hydrogen-deuterium substitution for neutrons have been developed in addition to the techniques introduced by Hauptmann and Karle for x-ray crystallography [1, 2] to overcome the phase-problem. Some of these methods are reviewed in a work by Taylor [3]. These methods, however, are in general only suitable for certain types of samples (e.g., crystals, non-light elements), or the sample has to be changed to perform the experiment, or both.

In polarized specular neutron reflectivity a method has been developed to obtain both amplitude and phase of the wave reflected from a thin film structure using a magnetic reference layer and a polarized neutron beam, enabling an unambiguous determination of the scattering length density (SLD) profile normal to the film surface [4–7]. In the method developed for specular neutron reflectometry, it was necessary to formulate a first-principles method in terms of a solution of the Schroedinger wave equation since the reflectivity at low values of wavevector transfer Q in the neighborhood of the critical angle for total external reflection can be sufficiently large that the typically applied Born approximation is no longer valid.

In the interest of further developing and possibly improving upon the basic concept of employing reference structures to extract phase information, a variation of such a technique was introduced [8] for dilute-solution small-angle neutron scattering (SANS) in which: (1) a reference object is attached externally to the sample object of incompletely known structure in a specific orientation and at a particular position; (2) two independent scattering experiments are then performed, one for an ensemble of composite objects with reference part ‘A’ and the other with reference part ‘B’—the sample part of each object being the same; and (3) the data is analyzed in terms of the unknown part of the sample structure rendered into finite elements of arbitrarily-sized volume. As was shown in [8], the incrementally different method for phase determination introduced therein recovers, in principle, the sought after phase information and, in addition, simultaneously retrieves information lost to orientational averaging of the sample objects because of their random orientations when suspended in a liquid environment. The latter orientational information can be extracted because there exists a fixed relationship between sample and reference in the specific type of configuration employed in this method.

In essence, the phase-sensitive method that is investigated experimentally here differs in one key aspect from a more conventional isomorphic substitution (in crystallography) or contrast variation (in small-angle scattering). Instead of isomorphically replacing atom ‘A’ at a known location within a unit cell with an atom of type ‘B’—or effectively changing the contrast or SLD of one portion of a system containing a ferromagnetic component through the use of polarized beams (see e.g., [9])—and performing a conventional analysis of the two composite system (unknown part plus reference segment) scattering data sets, the fundamental manner in which the composite system data are analyzed is reformulated as originally introduced in [8]. A synopsis of that reformulated analysis is given below as a more quantitative description of the basic idea.

**Figure 1.**
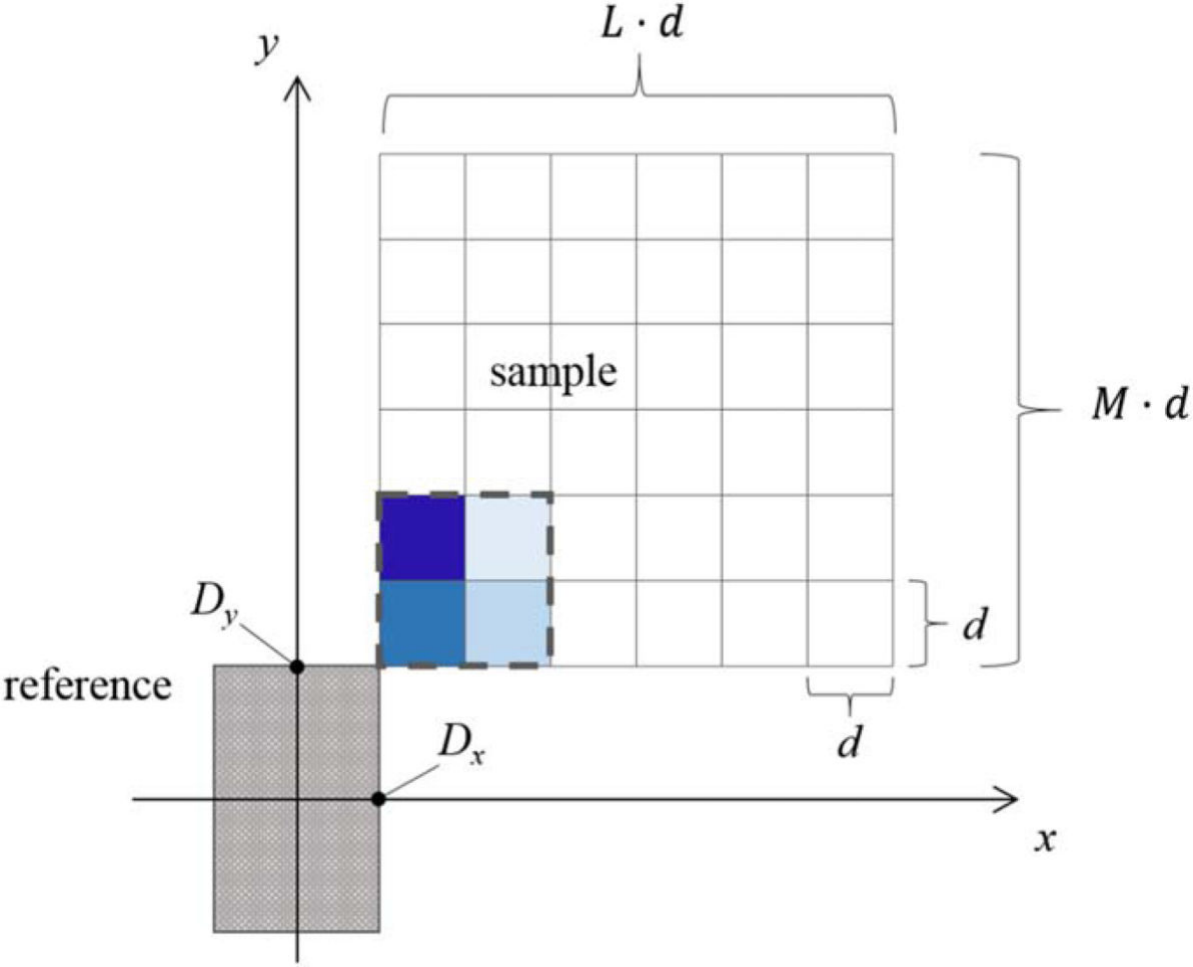
Two-dimensional composite structure depicted in which the unknown part of the structure is rendered in finite element form, each element having a constant SLD value. The reference part can be of any SLD distribution that satisfies the requisite requirements relating to symmetry and relative size (for a particular level of sensitivity) so long that it is completely known. In the example calculations in equations (9)–(16) only the first four squares are used but in general the sample is decomposed into L by M squares.

Imagine, for simplicity, the two-dimensional composite structure depicted in figure 1 in which the ‘unknown’ part of the structure is rendered in finite element form, the constant SLD values of each element to be determined through an analysis of two composite small-angle scattering data sets each corresponding to the common unknown part of interest plus one of two known reference parts. Then, following the original description in [8], we assume a collection of identical such objects, randomly oriented, in the dilute solution limit (negligible inter-particle correlations). The structure factor for any one of the identical objects in the ensemble, averaged over all possible angular orientations, is proportional to a differential scattering cross section. Note that we use the term ‘structure factor’ for the structure of an object as it is conventionally done in general scattering theory and not the term ‘form factor’ usually used in the small-angle scattering community. For the purposes of this discussion, we can neglect sample volume normalization factors and set the SLD of the solution to be zero. Prior to orientational averaging, the structure factor, $F_{\text{C}}$, as defined within the Born approximation, for a single composite two-dimensional object is given by

(1) $$F_{\text{C}}(\text{Q})=\iint_{yx}\rho (x,y)\exp\left[i\left(Q_{x}x+Q_{y}y\right)\right]dxdy,$$

where ρ(x,y) is the SLD to be determined. The integration is over the entire volume of the object (both unknown and reference parts) and the wavevector transfer Q and the position vector r are expressed in the object coordinate system (x,y). We can write the composite structure factor $F_{\text{C}}$ as the sum of two parts, one for the unknown part of the object and the other for the reference piece (this corresponds to the sum of two integrals, each performed over the respective partial volume):

(2) $$F_{\text{C}}(\text{Q})=F_{\text{R}}(\text{Q})+F_{\text{S}}(\text{Q}),$$

where the subscripts ‘R’ and ‘S’ denote reference and unknown ‘sample of interest’ parts of the composite object. In any scattering experiment, a scattered intensity is measured which is proportional to the complex square of the structure factor, which for a symmetric component $F_{\text{R}}(\text{Q})$ is given by

(3) $${\left|F_{\text{C}}\right|}^{2}={\left|F_{\text{R}}\right|}^{2}+{\left|F_{\text{S}}\right|}^{2}+2\text{Re}F_{\text{R}}\text{Re}F_{\text{S}},$$

since $F_{\text{R}}$ is real for a symmetric reference. Because of the random orientations of the objects in dilute solution, the expression in equation (3) must be averaged over the entire solid angle. Thus, denoting this orientational average by ‘〈〉’, we need

(4) $$\langle {\left|F_{\text{C}}\right|}^{2}\rangle =\langle {\left|F_{\text{R}}\right|}^{2}\rangle +\langle {\left|F_{\text{S}}\right|}^{2}\rangle +\langle 2\text{Re}F_{\text{R}}\text{Re}F_{\text{S}}\rangle .$$

Now suppose that, in principle at least, the reference part of the composite object could be replaced with a piece of identical size and shape but with a different uniform SLD (one possible way is to use a ferromagnetic reference part in conjunction with a polarized beam, as already mentioned). Two independent scattering experiments could then be performed, one for an ensemble of composite objects with reference part ‘A’ and the other with reference part ‘B’—the sample part of each object being the same ($F_{\text{R}}$ for either A or B could also be zero). The difference between the two data sets of scattered intensities thus collected is proportional to the difference in the corresponding orientationally-averaged square of the composite structure factors. Defining this difference function to be D(Q), the following relation can be written using equation (4):

(5) $$D(Q)\equiv \langle {\left|F_{\text{CA}}\right|}^{2}\rangle -\langle {\left|F_{\text{CB}}\right|}^{2}\rangle =\langle {\left|F_{\text{RA}}\right|}^{2}\rangle -\langle {\left|F_{\text{RB}}\right|}^{2}\rangle +2\langle \text{Re}F_{\text{RA}}\text{Re}F_{\text{S}}\rangle -2\langle \text{Re}F_{\text{RB}}\text{Re}F_{\text{S}}\rangle ,$$

where the subscripts ‘RA’ and ‘RB’ refer to the two different reference parts successively attached to the common sample part of the composite scattering object. Since the composite system scattered intensities can be measured and the reference quantities $\langle {\left|F_{\text{RA}}\right|}^{2}\rangle$ and $\langle {\left|F_{\text{RA}}\right|}^{2}\rangle$ can be computed, then the quantity

(6) $$U(Q)=\langle \text{Re}F_{\text{RA}}\text{Re}F_{\text{S}}\rangle -\langle \text{Re}F_{\text{RB}}\text{Re}F_{\text{S}}\rangle$$

can be directly solved for. Equation (6) is an implicit equation for the desired $\text{Re}F_{\text{S}}$ that is embedded within the orientational average as a product with the real part of the known quantities $\text{Re}F_{\text{RB}}$ and $\text{Re}F_{\text{RA}}$. In the special case where $\text{Re}F_{\text{S}}$ is isotropic, it can be taken out of the orientational average and directly solved for. For the general case, however, it is problematic to extract $\text{Re}F_{\text{S}}$ from the average in which it is embedded. Conventionally, in small-angle scattering the quantity F is defined to be the ‘form’ factor instead of the ‘structure’ factor used in crystallography and as is adopted here.

In SAS, the rotationally averaged square of F is typically analyzed in terms of a radius of gyration. However, ideally, $\text{Re}F_{\text{S}}$ itself is not what we are ultimately after but, rather, the function $\rho_{s}(\text{r})$ of which $F_{\text{S}}$ is the Fourier transform. With the aid of a piece-wise continuous representation of $\rho_{s}(\text{r})$ over a suitable mesh of cells, a set of discrete element values defining $\rho_{s}(\text{r})$ can be extracted, in principle, from the RHS of equation (6) through an algebraic rearrangement of terms and subsequent solution of a set of linear, simultaneous equations. This is the essential difference in the approach, as originally introduced in [8], that we are taking in the analysis of the SANS data and are illustrating here with the two-dimensional example represented in figure 1. In this case, the structure factor for the rectangular solid reference part of the object, $F_{\text{R}}$, centered on the composite object coordinate system, is given by

(7) $$F_{\text{R}}=\iint_{yx}\rho_{\text{R}}\exp\left[i\left(Q_{x}x+Q_{y}y\right)\right]dxdy=\left(4\rho_{\text{R}}/Q_{x}Q_{y}\right)\text{sin}\left(Q_{x}D_{x}\right)\text{sin}\left(Q_{y}D_{y}\right)=\text{Re}F_{\text{R}}$$

where the integration limits are from $-D_{x}$ to $+D_{x}$ and similarly in the y-direction. In the above expression, the dimensions of the rectangular reference are $2D_{x}$ and $2D_{y}$ as indicated in figure 1. (The uniform SLD of the reference, $\rho_{\text{R}}$, has units of inverse length squared.)

The structure factor for the sample part of the object has a more complicated form, one which can describe an arbitrary shape and SLD distribution, and is given by (where the integration limits are now from $D_{x}$ to $D_{x}+Ld$ and analogously in the y−direction):

(8) $$F_{\text{S}}=\iint_{yx}\rho_{s}(x,y)\exp\left(iQ_{x}x\right)\exp\left(iQ_{y}y\right)dxdy.$$

Explicit evaluation of the above expression for the 4-element square model of figure 1 gives

(9) $$F_{S}=\int_{Dy}^{Dy+d}\left[\int_{Dx}^{Dx+d}\rho (x,y)\exp\left(iQ_{x}x\right)dx+\int_{Dx+d}^{Dx+2d}\rho (x,y)\exp\left(iQ_{x}x\right)dx\right]\exp\left(iQ_{y}y\right)dy\;\;\;+\int_{Dy+d}^{Dy+2d}\left[\int_{Dx}^{Dx+d}\rho (x,y)\exp\left(iQ_{x}x\right)dx+\int_{Dx+d}^{Dx+2d}\rho (x,y)\exp\left(iQ_{x}x\right)dx\right]\exp\left(iQ_{y}y\right)dy.$$

Making explicit use of the finite element rendering of the sample part of the object, the equation immediately above can be rewritten as

(10) $$F_{\text{S}}=\rho_{11}\int_{Dy}^{Dy+d}\left[\int_{Dx}^{Dx+d}\exp\left(iQ_{x}x\right)dx\right]\exp\left(iQ_{y}y\right)dy+\rho_{21}\int_{Dy}^{Dy+d}\left[\int_{Dx+d}^{Dx+2d}\exp\left(iQ_{x}x\right)dx\right]\exp\left(iQ_{y}y\right)dy\;\;\;+\rho_{12}\int_{Dy+d}^{Dy+2d}\left[\int_{Dx}^{Dx+d}\exp\left(iQ_{x}x\right)dx\right]\exp\left(iQ_{y}y\right)dy+\rho_{22}\int_{Dy+d}^{Dy+2d}\left[\int_{Dx+d}^{Dx+2d}\exp\left(iQ_{x}x\right)dx\right]\exp\left(iQ_{y}y\right)dy,$$

where the $\rho_{ij}$ denote the constant values of SLD within the specific (i, j) finite element square. Then the real part of $F_{\text{S}}$ can be written, after performing the indicated integrations, as

(11) $$\text{Re}F_{\text{S}}=\left[4/\left(Q_{x}Q_{y}\right)\right]\text{sin}\left(Q_{x}d/2\right)\text{sin}\left(Q_{y}d/2\right)\;\;\;\;\times \{\rho_{11}\text{cos}\left[Q_{x}\left(D_{x}+d/2\right)+Q_{y}\left(D_{y}+d/2\right)\right]+\rho_{21}\text{cos}\left[Q_{x}\left(D_{x}+d/2+d\right)+Q_{y}\left(D_{y}+d/2\right)\right]\;\;\;\;+\rho_{12}\text{cos}\left[Q_{x}\left(D_{x}+d/2\right)+Q_{y}\left(D_{y}+d/2+d\right)\right]+\rho_{22}\text{cos}[Q_{x}\left(D_{x}+d/2+d\right)\;\;\;\;+Q_{y}\left(D_{y}+d/2+d\right)]\}.$$

Equation (6) can be written explicitly in terms of the angular average of all orientations θ of the composite object in two dimensions as

(12) $$U(Q)=\frac{1}{2\pi }\int_{0}^{2\pi }\left(\text{Re}F_{\text{RA}}-\text{Re}F_{\text{RB}}\right)\text{Re}F_{\text{S}}d\theta .$$

Substituting the expressions for $\text{Re}F_{\text{RA}}$, $\text{Re}F_{\text{RB}}$, and $\text{Re}F_{\text{S}}$ from equations (7) and (11), respectively, we obtain

(13) $$U(Q)=\rho_{11}\int_{0}^{2\pi }W_{11}(\theta )d\theta +\rho_{21}\int_{0}^{2\pi }W_{21}(\theta )d\theta +\rho_{12}\int_{0}^{2\pi }W_{12}(\theta )d\theta +\rho_{22}\int_{0}^{2\pi }W_{22}(\theta )d\theta ,$$

where the quantitites $W_{ij}$ are functions of the angle θ only. For instance, the explicit form of $W_{11}(\theta )$ is

(14) $$W_{11}(\theta )=[1/(2\pi )]\left[4\left(\rho_{\text{RA}}-\rho_{\text{RB}}\right)/\left(Q_{x}Q_{y}\right)\right]\text{sin}\left(Q_{x}D_{x}\right)\text{sin}\left(Q_{y}D_{y}\right)\;\;\;\;\times \left[4\pi /\left(Q_{x}Q_{y}\right)\right]\text{sin}\left(Q_{x}d/2\right)\text{sin}\left(Q_{y}d/2\right)\text{cos}\left[Q_{x}\left(D_{x}+d/2\right)+Q_{y}\left(D_{y}+d/2\right)\right].$$

The average over all composite orientations relative to the direction of an incident neutron wavevector is equivalent to an average over all directions of wavevector transfer Q so that substituting $Q_{x}=Q\text{cos}(\theta )$ and $Q_{y}=Q\text{sin}(\theta )$ allows the coefficients of the SLD values of the elemental squares of the unknown part of the object to be directly computed. Defining the coefficients of the $\rho_{ij}$ as

(15) $$C_{ij}\int_{0}^{2\pi }W_{ij}(\theta )d\theta .$$

With L and M elemental squares along the x- and y–axis respectively (see figure 1), we finally arrive at a system of linear equations for LM unknowns $\rho_{ij}$ describing the finite element representation of the SLD distribution of the unknown part of the composite object—with coefficients which can be calculated and values of the U(Q) which can be determined from two independent scattering measurements

(16) $$U\left(Q_{1}\right)=\rho_{11}C_{11}\left(Q_{1}\right)+\rho_{21}C_{21}\left(Q_{1}\right)+\rho_{12}C_{12}\left(Q_{1}\right)+\rho_{22}C_{22}\left(Q_{1}\right)\;U\left(Q_{2}\right)=\rho_{11}C_{11}\left(Q_{2}\right)+\rho_{21}C_{21}\left(Q_{2}\right)+\rho_{12}C_{12}\left(Q_{2}\right)+\rho_{22}C_{22}\left(Q_{2}\right)\;U\left(Q_{3}\right)=\rho_{11}C_{11}\left(Q_{3}\right)+\rho_{21}C_{21}\left(Q_{2}\right)+\rho_{12}C_{12}\left(Q_{3}\right)+\rho_{22}C_{22}\left(Q_{3}\right)\;U\left(Q_{4}\right)=\rho_{11}C_{11}\left(Q_{4}\right)+\rho_{21}C_{21}\left(Q_{2}\right)+\rho_{12}C_{12}\left(Q_{4}\right)+\rho_{22}C_{22}\left(Q_{4}\right).$$

As shown in detail in [8], the 2D solution outlined above to illustrate the basic concept of this particular phase-sensitive method can be generalized to three dimensions and that a variety of different reference structures can be employed, including, for example, ferromagnetic materials in conjunction with polarized beams. In the main body of this paper we present the first experimental realization of this particular phase-sensitive small-angle neutron scattering (PS-SANS) method on a simple test system consisting of magnetic iron oxide particles (reference) with an unknown polymer shell (sample).

## 2. An initial application of the general method to a simpler spherically symmetric system

Our test system consists of a magnetic iron oxide (Fe_3_O_4_) particle core and a polymer shell of unknown composition and thickness. The magnetic core, with known radius $R_{\text{M}}$ and $SLD\rho_{\text{M}}$, serves as the reference for PS-SANS and the sample with an SLD distribution to be determined is the nuclear scattering part of the core and the polymer shell. The theoretical basis of PS-SANS is described in detail in Majkrzak *et al* [8] and illustrated in the 2D example above. Here we will employ the same formalism but with the simplifications that arise because of the spherical symmetry of our system.

Assuming that the system is in the dilute solution limit the structure factor of one compound particle (sample + reference) is given by equation (2). In a diffraction experiment, the intensity of the scattering from the compound object will be proportional to ${\left|F_{\text{C}}\right|}^{2}$. Because of the spherical symmetry of sample and references no orientational averaging is needed, and both $F_{\text{S}}$ and $F_{\text{R}}$ are real. The difference between the two measurements will then be

(17) $$D(Q)\propto {\left|F_{\text{RA}}\right|}^{2}-{\left|F_{\text{RB}}\right|}^{2}+2\left(F_{\text{RA}}F_{\text{S}}-F_{\text{RB}}F_{\text{S}}\right),$$

where the subscripts A and B refer to the two measurements with different references. If the reference is a ferroor ferrimagnetic particle magnetized along some direction and two measurements are performed with neutron polarization parallel and antiparallel to the magnetization respectively, then the same sample-reference system can be used for the two measurements.

In our case the reference is a purely magnetic scatterer (the nuclear scattering of the particle core is considered part of the sample) and $F_{\text{RA}}$ and $F_{\text{RB}}$ differ only in the sign of the (magnetic) SLD, so $F_{\text{RA}}=-{\text{F}}_{\text{RB}}\equiv {\text{F}}_{\text{R}}$ and consequently

(18) $$D(Q)=4F_{\text{R}}F_{\text{S}}=2U(Q).$$

The structure factor of the reference can be calculated and in our case it will be the familiar structure factor of a sphere with radius $r=R_{M}$. The structure factor for a spherical particle with radius r is

(19) $$F_{\text{Sph}}\left(Qr\right)=4\pi r^{3}\frac{j_{1}\left(Qr\right)}{Qr},$$

where $j_{1}$ is the spherical Bessel function of the first kind.

**Figure 2.**
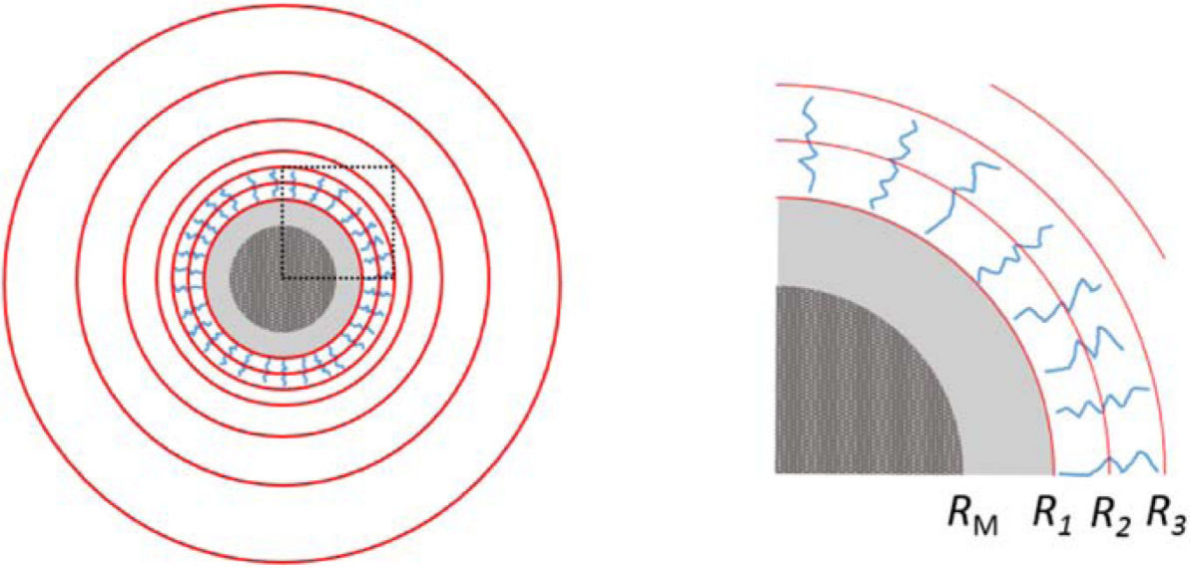
Finite element decomposition: the sample structure, which consists of an iron oxide core and a polymer shell, is described by the SLD in a spherical core with radius $R_{1}$ and N−1 concentric shells of varying thickness. $R_{\text{M}}$ is the radius of the magnetic reference. The red concentric circles in the figure represent the FED. The sample is generally unknown and the FED dimensions do not necessarily correspond to the sample dimensions. The relative sizes of features in the schematic drawing do not represent actual dimensions of the sample or the FED used in the inversion. The area in the dashed square on the left part of the figure is enlarged in the right part of the figure.

The unknown sample structure can be described by decomposing the continuous SLD distribution into a finite number of elements. In the case of an asymmetric sample a regular grid of cubic elements of equal volume would be an obvious choice. However, because of the spherical symmetry of our system the SLD only varies in the radial direction and it is advantageous to describe the structure using a finite element decomposition (FED) that reflects this symmetry. The FED we will use here is a homogeneous sphere with radius $R_{1}$ and a number of concentric spherical shells of constant SLD as depicted in figure 2. The individual shells need not have the same thickness (or volume) and furthermore, the magnetic and nuclear core does not need to have the same size ($R_{1}\neq R_{\text{M}}$ in general). For an FED consisting of a spherical core and N−1 shells, the measured nuclear-magnetic cross term can be described as

(20) $$U(Q)=\frac{2\text{Φ}}{V_{\text{tot}}}\rho_{\text{M}}F_{\text{sph}}\left(QR_{\text{M}}\right)\sum_{j=1}^{N}\rho_{j}F_{j}(Q),$$

where Φ is the volume fraction of core–shell particles in the solvent and $V_{\text{tot}}$ is the total FED volume $\left(4\pi R_{N}^{3}/3\right)$. For the j'th element of the FED $\rho_{j}$ is the SLD and $F_{j}(Q)$ is the structure factor, i.e., the first term, corresponding to the core is simply

(21) $$F_{Q,1}=F_{\text{sph}}\left(QR_{1}\right)$$

and the remaining coefficients corresponding to the shells are

(22) $$F_{Q,j}=F_{\text{sph}}\left(QR_{j}\right)-F_{\text{sph}}\left(QR_{j-1}\right)\;\text{for}\;j⩾2,$$

where $R_{j}$ is the outer radius of shell number j−1.

If U(Q) is measured in $N_{Q}Q$-points, equation (20) is a linear system of $N_{Q}$ equations in N unknowns that can conveniently be expressed as a matrix equation

(23) $$\left(\begin{array}{c} U\left(Q_{1}\right)\\ \vdots \\ U\left(Q_{N_{Q}}\right) \end{array}\right)=\left(\begin{array}{ccc} C_{1}\left(Q_{1}\right) & \cdots & C_{N}\left(Q_{1}\right)\\ \vdots & \ddots & \vdots \\ C_{1}\left(Q_{N_{Q}}\right) & \cdots & C_{N}\left(Q_{N_{Q}}\right) \end{array}\right)\left(\begin{array}{c} \rho_{1}\\ \vdots \\ \rho_{N} \end{array}\right).$$

Equation (23) can be solved for the unknown $\rho_{j}\text{s}$ using numerical methods. Here we use the *pinv* function in MATLAB^7^ which uses a truncated singular value decomposition (SVD) to calculate a pseudoinverse of the coefficient matrix. An advantage of this method is that a parameter *tol* can be chosen so that all singular values smaller than *tol* are treated as zero. Choosing a suitable *tol* stabilizes the results because it effectively suppresses experimental noise as well as round-off errors and other numerical artifacts. For comparison, results using QR-decomposition (through MATLABʼs ⧹ operator) are also briefly discussed.

### 2.1 Resolution smearing

When inverting experimental data it is necessary to take the finite Q—resolution of the instrument into account. This is done by smearing each of the $C_{j}$ coefficients of equation (23) with the resolution function $R\left(Q^{\prime },Q\right)$

(24) $$C_{j,\text{smeared}}(Q)=\int_{0}^{\infty }R\left(Q^{\prime },Q\right)C_{j}(Q)dQ^{\prime }.$$

The resolution function is well approximated by a normalized Gaussian with a σ that can be calculated for each Q − *point* [10, 11]. Size polydispersity of the reference can be treated in exactly the same way as the instrumental resolution, but with the resolution function replaced by the polydispersity function [12].

## 3. Sample characterization

**Figure 3.**
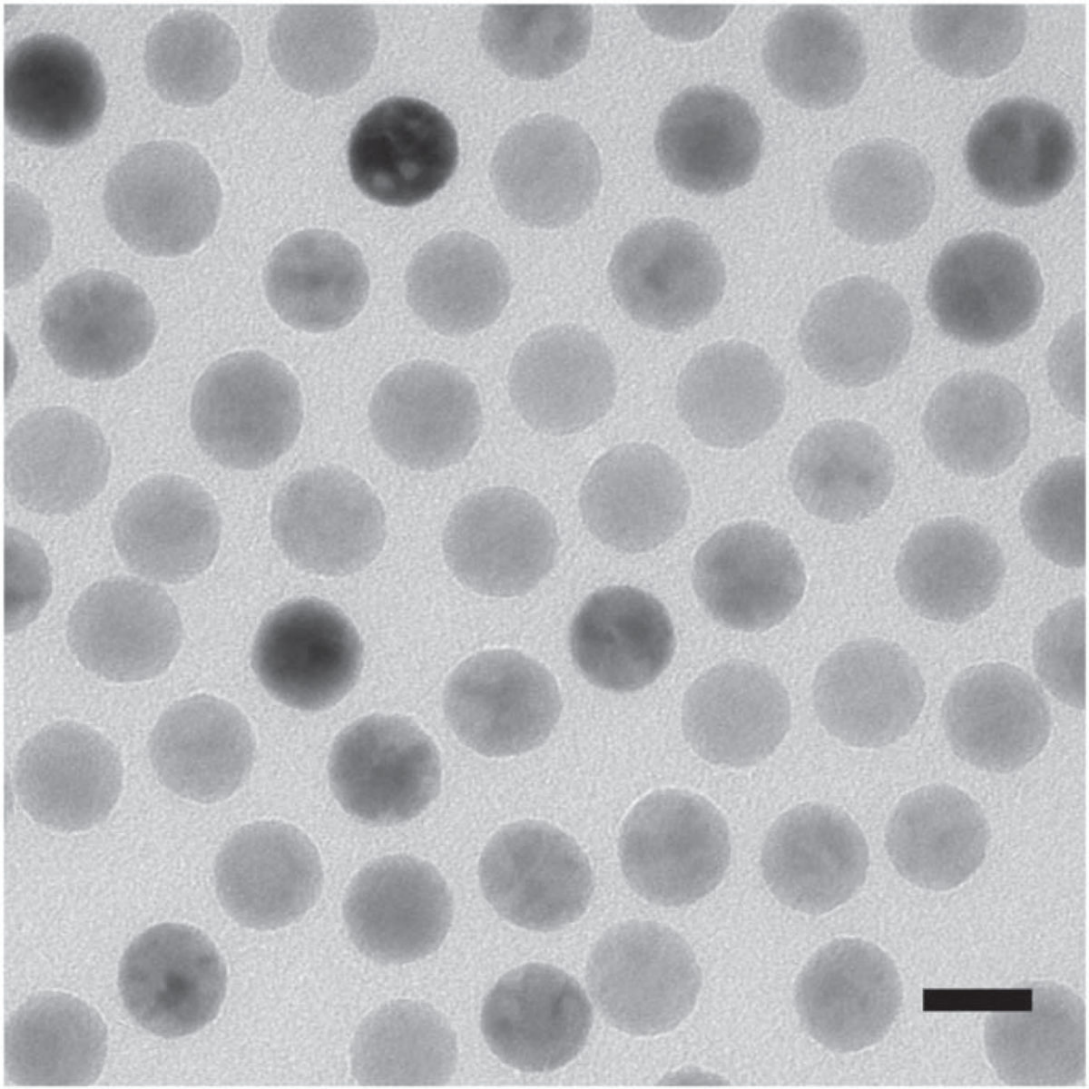
Transmission electron microscopy image of iron oxide particle cores. The scale bar is 25 nm.

The PrecisionMRX^®^ core–shell particles for PS-SANS were produced by Imagion Biosystems for use in magnetic relaxometry, an experimental technique for the diagnosis of cancer [13]. The particles consist of Fe_3_O_4_ cores coated with a monolayer of oleic acid, a monolayer of amphiphilic polymer and a layer of polyethylene glycol. The cores are produced with a diameter of 25 nm and a Gaussian size distribution with a polydispersity smaller than 10% [14]. The uniformity in size and shape of the cores is confirmed by transmission electron microscopy as seen in the image in figure 3. High-resolution transmission electron microscopy and powder x-ray diffraction confirm that the cores consist of Fe_3_O_4_ with no indications of other phases [14]. Magnetometry data shows that the magnetization of the cores is almost fully saturated at an applied magnetic field of 100 mT and reaches a saturation magnetization of about 70 Am^2^/kg Fe_3_O_4_ [13–15] corresponding to 3.6 · 10^5^ A/m which is about 75% of the value for bulk magnetite.

The sample for PS-SANS experiments consisted of core–shell particles suspended in D_2_O at an Fe concentration of 10 mg/mL corresponding to a volume fraction of 0.0019. For the experiment, approximately 0.8 mL of sample was filled in a standard 2 mm-path length titanium cell with quartz windows.

A sample of dry iron oxide cores was produced from oleic acid covered iron oxide cores suspended in H_2_O by dripping it onto aluminum foil and allowing the solvent to evaporate. The drying resulted in a sticky powder that was kept in the aluminum foil and used for polarization analysed SANS measurements (PASANS).

### 3.1. Polarization analysed SANS characterization of reference

To characterize the magnetic core to be used as reference in PS-SANS, a PASANS experiment was performed on the sample of dried iron oxide cores. The measurements were performed at the NG7-SANS instrument at NIST Center for Neutron Research (NCNR) using a polarized neutron beam and a polarized ^3^He analyzer. The sample was placed in zero applied field, except for the small guide field (≈2 mT) necessary to maintain the neutron polarization.

**Figure 4.**
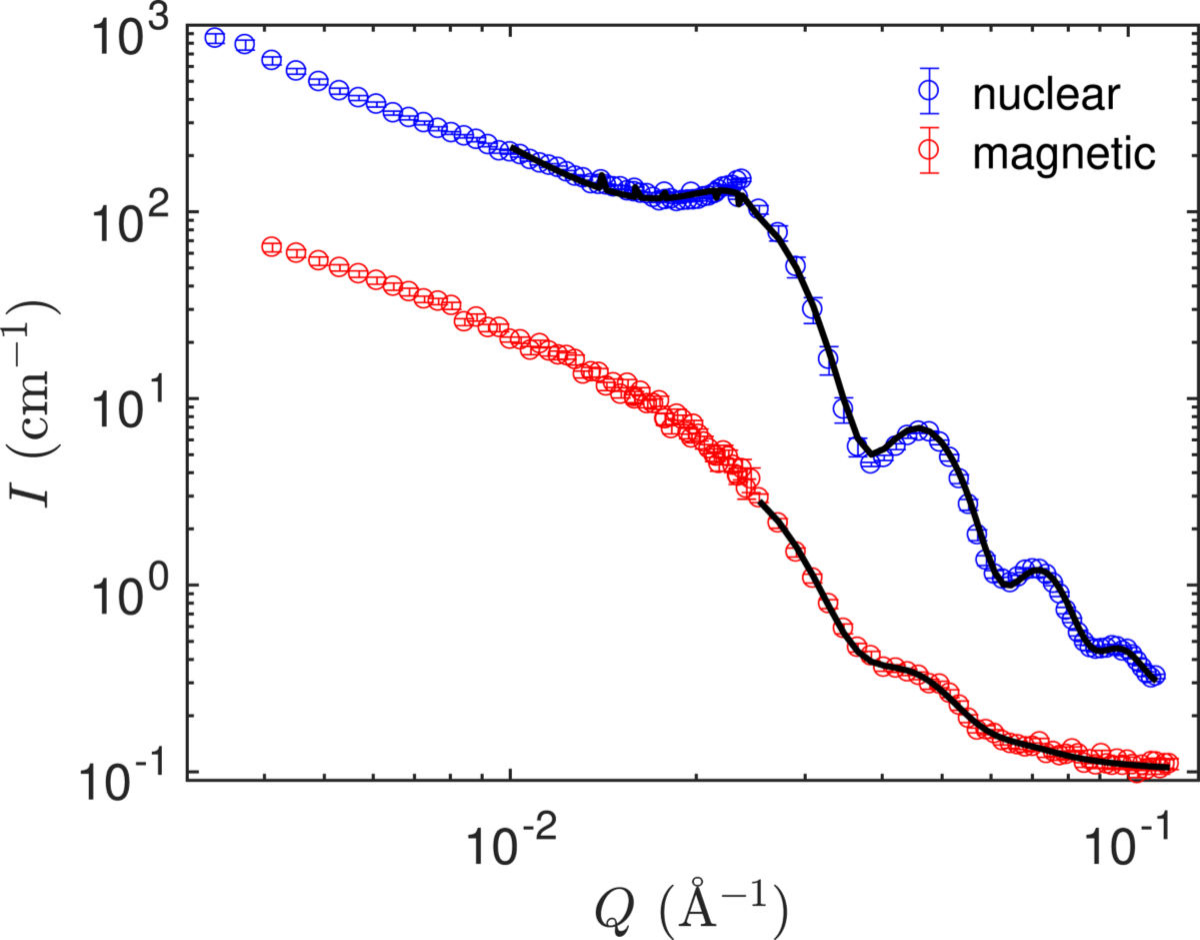
PASANS data from dried particle cores. The nuclear signal is determined from the non-spin-flip scattering wheras the magnetic signal is determined from the spin-flip scattering and corresponds to the magnetization perpendicular to the guide field. The full lines are fits to an FCC paracrystal model. The error bars represent one standard deviation.

With PASANS it is possible to separate the magnetic from the nuclear scattering, allowing us to characterize the magnetic scattering of the particles. The magnetic and nuclear SANS data is shown in figure 4. The nuclear signal is determined from the non-spin-flip scattering parallel to the applied field and the magnetic signal is determined from the spin-flip scattering perpendicular to the applied field and comes from the magnetization perpendicular to the applied (guide) field. For more information about PASANS see [16–19].

Our main objective here is to characterize the magnetic scattering of the cores. The structure of the individual particles can be extracted from the higher-Q part of the scattering while the scattering at low Q reflects the packing of the powder. To take the packing of the particles into account both the nuclear and magnetic signal was fitted to a face-centered-cubic (FCC) paracrystal model [20, 21] using the Sasview software [22]. The scattering at low-Q could not be captured satisfactorily with the FCC model, which indicates that the packing of the particles is not perfectly described by the FCC structure. To obtain the optimal fit of the high-Q region only the $Q>0.025\;{\text{Å}}^{-1}$ region of the magnetic signal was included in the fit. For the nuclear signal data with $Q>0.01\;{\text{Å}}^{-1}$ was included in the fit. In both cases the model represents the data well in the fitted Q-range and captures the important features, such as the high-Q oscillations from the particle dimensions and the correlation peak at $Q\approx 0.023\;{\text{Å}}^{-1}$ in the nuclear scattering.

The nuclear and magnetic particle sizes determined from the fits are given in table 1. The nuclear radius of 12.50 (2) nm corresponds exactly to the nominal 25 nm particle diameter and the 4.6 (2) % polydispersity confirms the narrow size distribution. The magnetic size comes out smaller than the particle size with a radius of 11.8 (2) nm consistent with a 0.7 nm surface layer that is either magnetically disordered or has a different order than the bulk of the particles. Magnetic disorder near the surface of magnetic nanoparticles has been proposed by many authors as explanation of the reduced magnetization of magnetic nanoparticles compared to bulk materials [23, 24], although direct experimental observations of the disordered layer are more scarce. At 13.8 % the polydispersity of the magnetic size is significantly larger than the narrow size distribution of the particles.

There is some variation in the results obtained from analysis of the PASANS data, depending on how the parameters are constrained, e.g., whether or not the FCC packing is required to be the same in the nuclear and magnetic data. From these variations, we can deduce that the radius of the core is in the range 118–121 Å with a polydispersity in the range 0.12–0.15. The values given in table 1 are the ones giving the best fit.

## 4. PS-SANS experiment

**Figure 5.**
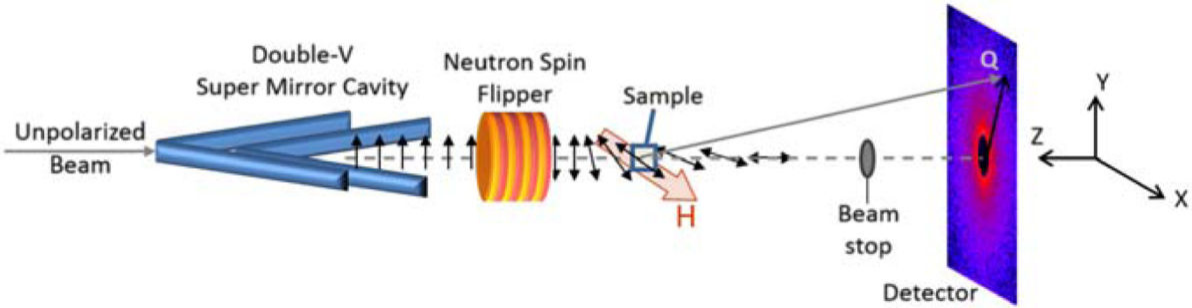
Experimental setup for polarized SANS. The ↑ neutrons are selected by the double-V supermirror cavity and the polarization is either left unchanged or rotated by 180◦ (flipped) depending onwhether the neutron spin flipper is off or on. A guide field covers the entire path from polarizer to sample and maintains the neutron polarization. The polarization of the scattered neutrons is not analyzed.

The PS-SANS experiment was performed at the NG7-SANS instrument at NCNR using 5.5 Å neutrons with a wavelength spread of 11.5% (FWHM) and two different sample-detector distances of 4.547 m and 12.547 m, resulting in a Q-range from 0.008 Å^−1^ to 0.077 Å^−1^. The initial spin state was prepared as ↑ or ↓ by a polarizing FeSi double-V supermirror and an electromagnetic spin-flipper. The sample was placed at room temperature in an electromagnet producing a 100 mT magnetic field along the (horizontal) x-direction which is enough to saturate the magnetization of the magnetically ordered core. Scattering of ↑ and ↓ neutrons were measured at each detector distance for one hour resulting in a total of 4 h of measurement time. The experimental setup is shown schematically in figure 5.

The nuclear-magnetic cross term U(Q) is obtained from the difference between scattering of ↑ and ↓ neutrons in the y-direction, i.e.


(25) $$2U(Q)=I_{y}^{↓}-I_{y}^{↑}.$$

$I_{y}^{↑}$ and $I_{y}^{↓}$ are determined from area-normalized sector slices with opening angles $\pm 15^{\circ }$ with respect to the vertical direction.

Incoherent scattering and any nuclear scattering that is uncorrelated to the magnetism, e.g. solvent scattering or scattering from excess polymer in the suspension, will scatter evenly in the ↑ and ↓ channels and will thus automatically be subtracted when U(Q) is calculated. Consequently, the only data reduction that was done consisted of masking out corrupted pixels near the edge of the detector, removing contaminated data points very close to the beam stop, normalizing the intensities to the individual detector efficiencies, and scaling the data to the sample transmission to obtain the intensity on an absolute scale. The data reduction was done using the Igor Pro macros developed at NCNR [25]. The reduction software automatically computes the instrumental resolution in each measured Q-point to be used for resolution smearing.

**Figure 6.**
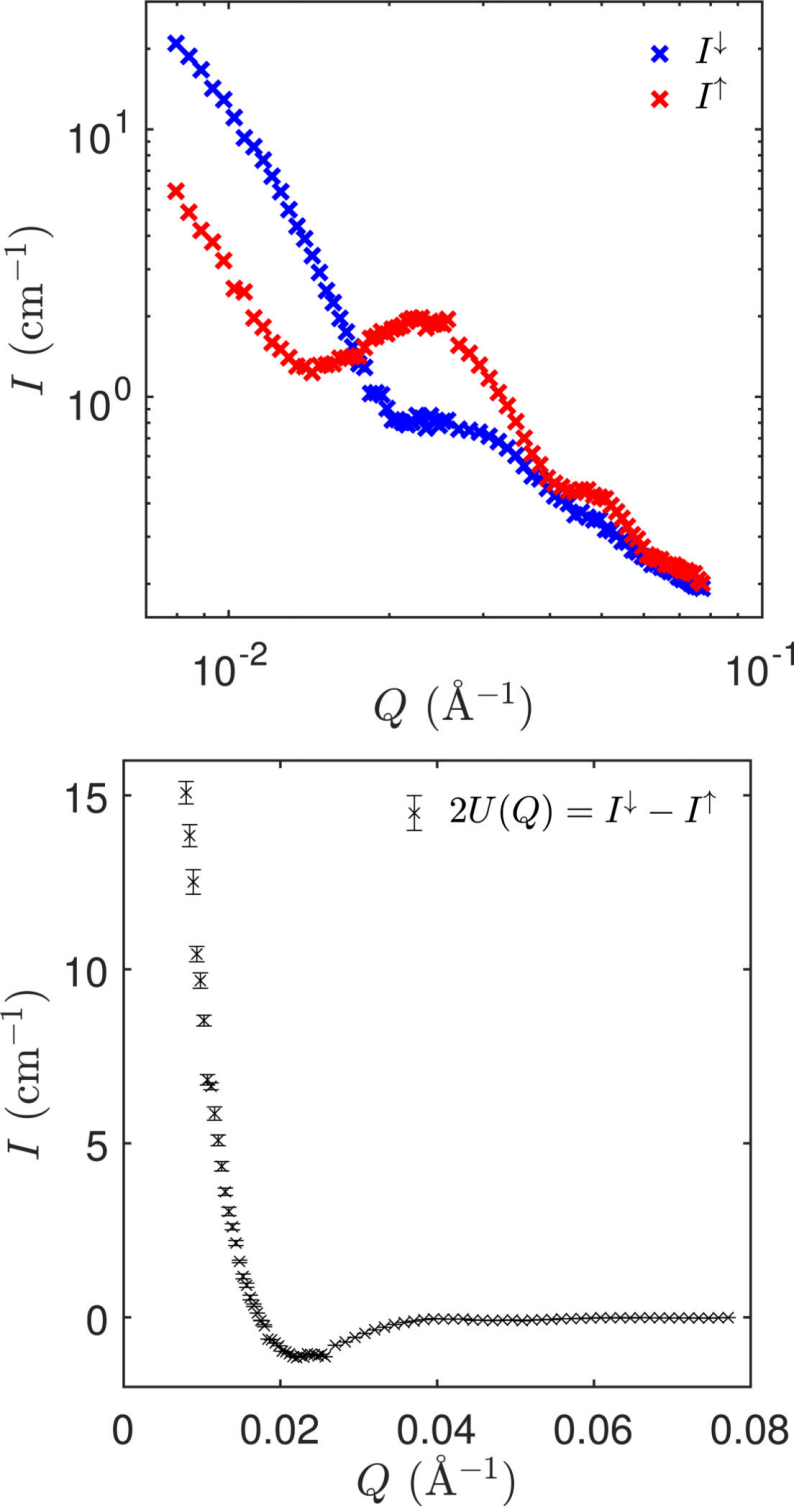
Polarized SANS data on core–shell nanoparticles in D_2_O. Top panel: measured intensities in the y-direction for the two spin states (note the logarithmic scale). The experimental uncertainty (standard deviation) is smaller than the size of the points. Bottom panel: measured cross term. The error bars represent one standard deviation.

The measured polarized SANS data is displayed in figure 6. The top panel shows the measured cross sections for ↑ and ↓ neutrons in the y-direction and the bottom panel shows the measured cross term U(Q).

## 5. Inversion of experimental data

### 5.1. Optimal resolution

The optimal spatial resolution, i.e., the smallest possible feature that can be distinguished in a diffraction experiment, is limited by the largest scattering vector $Q_{\text{max}}$ at which scattering is measured. When the phase is known the relationship between $Q_{\text{max}}$ and the finest lengthscale l that can be resolved is $l\approx \pi /Q_{\text{max}}$ and the resolution is thus twice as good as in a conventional diffraction experiment where $l\approx 2\pi /Q_{\text{max}}$ [7]. With $Q_{\text{max}}=0.077{\text{Å}}^{-1}$ we should expect an ideal resolution of l≈4.1nm in our experiment.

### 5.2. Finite element decomposition

To invert the experimental data we have to select the elements with which to describe the sample structure, i.e., define the radii of the core and shells in the FED. An obvious choice would be $R_{1}=12.5\;\text{nm}$ so the core in the FED corresponds to the known size of the particle cores. The thicknesses and number of shells in the FED should be chosen so that they correspond to the assumed sample dimensions (shell thickness of a few nanometers) and to the information content in the collected data.

To carry meaningful information the elements of the FED should not be finer than l=4.1nm. For the core radius this requirement is $2R_{1}\geq l$ which is well met when $R_{1}=12.5\;\text{nm}$, and for the shells the requirement is similarly that $2t_{j}≳l$, where $t_{j}$ is the thickness of the j’th shell. Note that the requirement is that the diameter, not the radius, of the feature to be resolved is larger than l. Consequently the shells in our FED should be no thinner than approximately 2 nm.

If perfect data was collected for $0<Q<Q_{\text{max}}$ and the total sample diameter was L, then the maximal number of free parameters in a model independent inversion could be quantified by the Nyqist number which can be determined as the integer part of $Q_{\text{max}}\text{L}/\pi$. However, because the data is not perfect the actual number of permissible free parameters in a model independent inversion could be smaller. For a given data set a suitable FED can be determined by trial and error. A poor choice of FED will be reflected in artifacts like unrealistic absolute values of the inverted SLDs or unphysical oscillations in the SLD profile.

### 5.3. Inverted SLD

The inversion was performed using a radius of 11.8 nm with 12.6% polydispersity for the reference. The reason for using a polydispersity of 12.6% and not 13.8% was that it slightly improved the stability of the inversion results. The used polydispersity is within one standard deviation of the result obtained with PASANS (see table 1).A (magnetic) SLD of $\rho_{\text{M}}=1.46\cdot 10^{-6}\;{\text{Å}}^{-2}$, corresponding to a magnetization of 5.12 · 10^5^ A/M was used.

**Figure 7.**
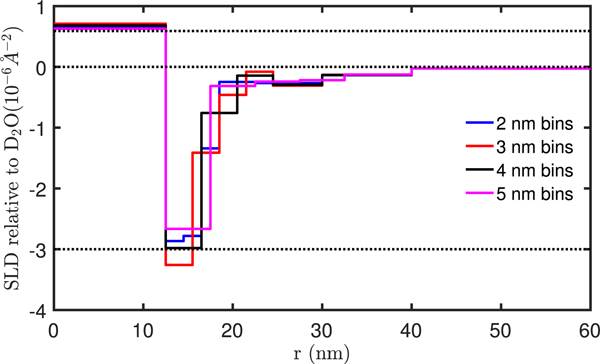
Inverted scattering length density profiles for different binnings. The SLDs are given relative to theD2Osolvent. The dashed line at a SLD of 0.59 ・10^−6^ Å^−2^ represents the expected SLD for bulk Fe3O4, the line at zero represents the solvent, and the line at −3 ・ 10^−6^ Å^−2^ is a guide for evaluating the SLD of the polymer shell. The outer radius of each shell is given in table 3.

Examples of inverted SLD profiles are shown in figure 7. The inverted SLDs are given relative to the D_2_O solvent. While the relative SLD expected for the iron oxide core is 0.59 · 10^−6^ Å^−2^, the polymer SLD is unknown. Typical SLDs for hydrogenated polymers are close to the SLD of H_2_O or slightly higher (up to $\approx 1.5\cdot 10^{-6}\;{\text{Å}}^{-2}$) while deuterated polymers have SLDs close to that of D_2_O [26, 27]. Depending on the degree of hydrogen-deuterium substitution in the solvent and on swelling of solvent molecules into the shell we can expect the SLD of the shell to be somewhere in between these values, i.e. between −6.94 · 10^−6^ Å^−2^ and 0 relative to D_2_O. The relevant SLDs are listed in table 2.

To resolve the structure of the polymer shell the inner shells are chosen to be thinnest and the outer shells thickest. The four inverted SLD profiles in figure 7 represent different FEDs, with inner shell thicknesses (or binnings) of 2 nm, 3 nm, 4 nm and 5 nm. The outer radii of each shell in the FEDs of figure 7 are shown in table 3. In all cases it was necessary to have the sample volume (the FED) extend to approximately 60 nm to obtain a reasonable inversion. For all four binning choices the SLD of the iron oxide core is reproduced reasonably well with an average core SLD of 0.67 (4) 10^−6^ Å^−2^, where the uncertainty is the deviation of the values determined with the four different binnings. In the first shell the SLD falls to ≈−3 10^−6^ Å^−2^ in all cases, indicating the change in the SLD from iron oxide to polymer. The SLD then increases first rather abruptly and then more gradually as it approaches the point of zero contrast (solvent).

**Figure 8.**
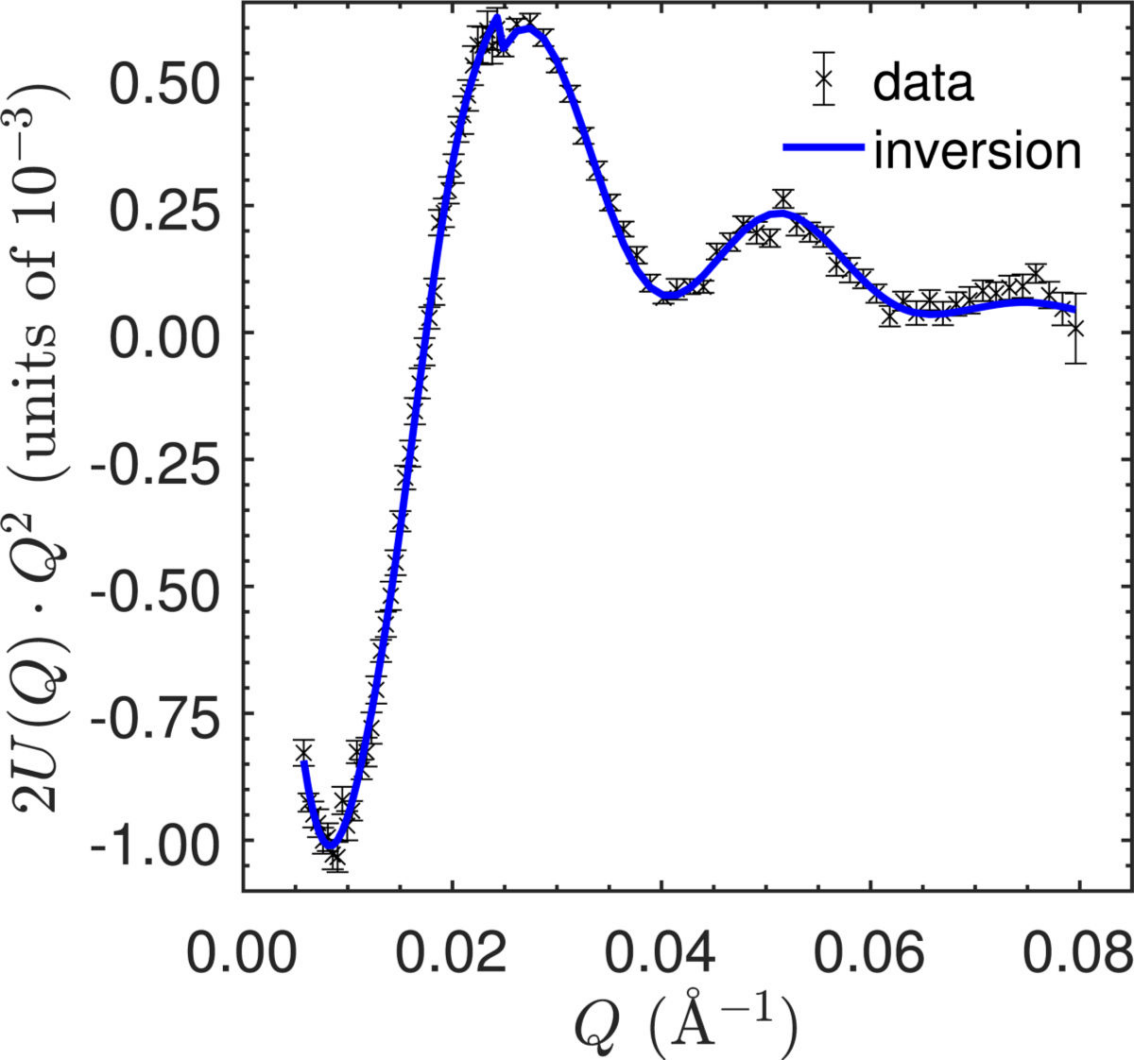
Comparison of the measured cross term U(Q) with the cross term calculated from equation (23) using the SLDs from the inversion with 2 nm bin size (and the corresponding radii from table 3). The data have been multiplied by $Q^{2}$ to accentuate features at higher Q. The error bars represent one standard deviation.

To verify that the inverted SLDs are in fact a solution to equation (23), U(Q) was calculated from the inverted SLDs and with the corresponding FED. This is shown in figure 8 for the inversion with 2 nm binning. The inverted U(Q) agrees well with the experimental, indicating a good solution. The results for the other binning choices are virtually indistinguishable.

Apart from relatively small differences in the exact size of the SLD the results obtained with the different binnings agree quite well. This shows that the results are robust with respect to changes in FED as long as a reasonable number of parameters is chosen. In our case it turned out that the maximum number of parameters in the FED was somewhere between 9 and 11 (8–10 shells). If more shells were used artificial oscillations appeared in the SLD profiles. A binning size of only 1 nm was also tried and while the results to some extent agree with the SLD profiles in figure 7, the results were very susceptible to small changes in the binning choices and the results were considered unreliable.

An SVD tolerance of $tol=10^{4}$ was chosen for the inversions presented here. In most cases $tol=10^{4}$ and tol=0 gave the same results. In the latter case the precision of the SVD inversion is determined by the machine epsilon (roundoff-error). The only exception was the inversion with a 2 nm binning, where tol=0 lead to oscillations in the inverted SLD profile. We also performed the inversion using QR decomposition which gave results identical or very similar to those obtained using SVD.

### 5.4. Sensitivity to changes in magnetic structure

**Figure 9.**
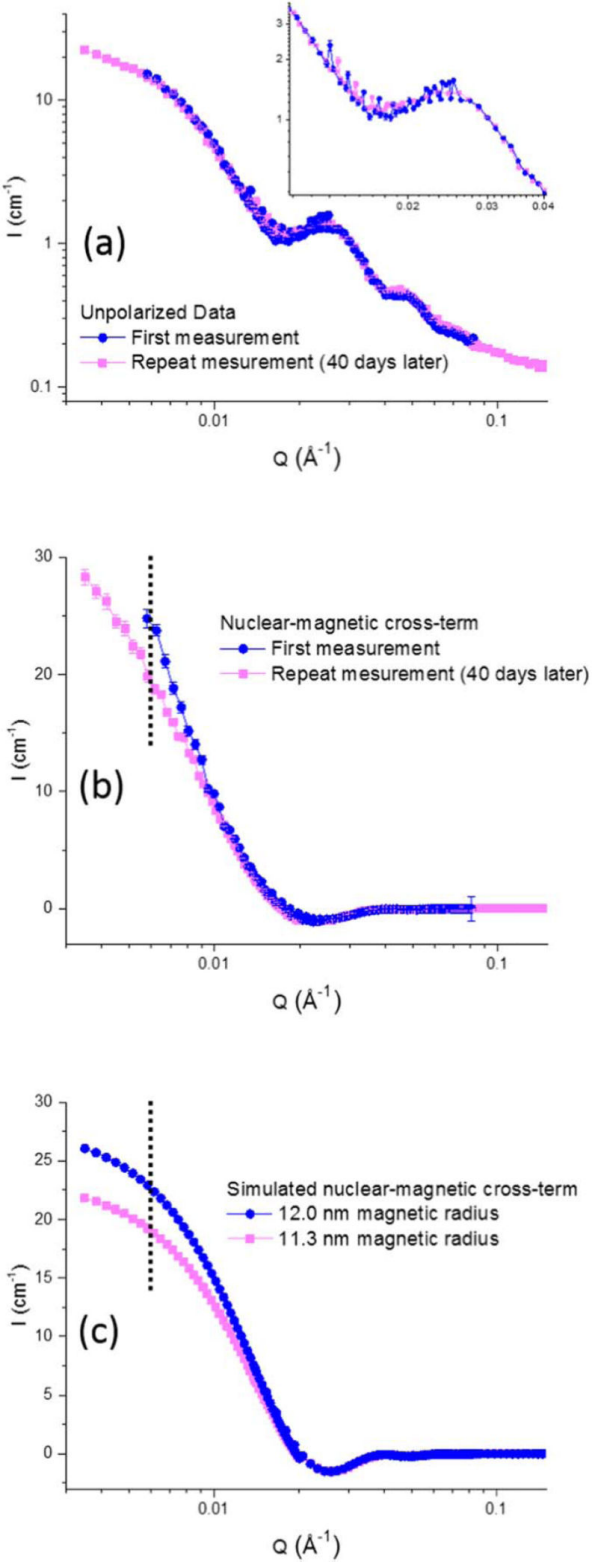
(a) Predominantly structural scattering from unpolarized SANS and (b) nuclear-magnetic cross-term taken from polarized scattering perpendicular to the applied magnetic field, where the scattering of the aged sample has been uniformly multiplied by a factor of 1.3. The inset in (a) emphasizes the close agreement in the predominantly structural scattering, where the oscillation in data points arises from an overlap in detector distances. (c) Simulation of single nanoparticles differing only in magnetic core size (but not structural core size). The dotted vertical line in (b, c) shows the lowest Q point common to both data sets for ease of comparison between experimental and simulated data splitting.

PS-SANS is highly sensitive to subtle magnetic changes, such as from sample aging. As illustration, we show the comparative measurements from our nanoparticles solvated in D_2_O shortly after their synthesis and 40 days later. Except for a global reduction in intensity of 33%, the predominantly structural scattering obtained from unpolarized SANS looks indistinguishable in shape (figure 9(a)). This means that apart from the probable precipitation of some particles from solution, the structural morphology and particle-to-particle distribution/agglomeration remains unaltered.

The nuclear-magnetic cross-term, however, shows pronounced differences (figure 9(b)). A uniform reduction in magnetic SLD across the magnetic cores would lead to a simple scaling reduction between the fresh and aged particles, which is not supported by experimental data in which the scattering difference is most pronounced at low-Q. Instead, the change can be qualitatively modeled by a reduction in magnetic core size, which could occur if aging reduces the magnetization per volume primarily at the Fe_3_O_4_ surface of the nanoparticles.

A simulation of single-particle, 12.5 nm Fe_3_O_4_ cores with a 4.0 nm thick polymer shell residing in D_2_O with either 12.0 nm or 11.3 nm magnetic cores is shown in figure 9(c). The D_2_O and Fe_3_O_4_ SLDs are set equal to bulk material values, while the polymer shell set 1.35 · 10^−6^ Å^−2^. As evidenced by the dotted vertical line at 0.006 Å^−1^ in figures 9(b)–(c), this reduction in magnetic core size can explain the low-Q divergence in scattering profiles between the fresh and aged samples, while maintaining close agreement in scattering profiles at higher Q. It is also apparent that the samples in both cases are not single-particle since the low-Q turnover from single-particle simulation is lacking in the data.

The point here is not to rigorously fit the aged data, but rather to demonstrate that PS-SANS can be used to detect magnetic differences between samples which would be challenging to detect with conventional SANS.

## 6. Discussion

Inversion of the PS-SANS data results in an SLD profile that corresponds well to that expected for the core–shell nanoparticles. The inverted core SLD of 0.67 (4) 10^−6^ Å^−2^ is 12% larger than expected for iron oxide. Furthermore, a magnetic SLD of 1.46 · 10^−6^ Å^−2^ corresponding to the saturation magnetization of bulk Fe_3_O_4_ was used in the inversion and not the lower magnetization expected for the nanoparticles. The expected magnetic SLD is thus 75% of the value used in the inversion, and accordingly the inverted SLDs should be rescaled by a factor of 1.33 giving a larger deviation from the expected SLD of iron oxide (almost 50%). This relatively large discrepancy can be explained by inaccuracies in the particle concentration (ϕ), or in the determined size or polydispersity of the reference. Considering that our method is model free and employs no scaling we consider it a success that we reproduce the iron oxide SLD so closely.

The SLD profile drops to a contrast of about −3 · 10^−6^ Å^−2^ and tapers off towards zero consistent with a well defined polymer shell extending approximately 4 nm from the core and a more loosely associated structure extending to about 40 nm (27.5 nm from the core). The inversion was only successful when the SLD profile was extended to about 60 nm or longer, indicating structural correlations significantly longer than the expected shell thickness.

The change in the nuclear-magnetic cross term for the samples aged for 40 days shows that the technique is very sensitive to changes in the magnetic structure of the reference. This shows that measuring the magnetic cross term can be used to study magnetic details that does not produce any significant change in the unpolarized scattering pattern. Furthermore, it shows that having a well characterized reference is of crucial importance for the PS-SANS method to work.

**Figure A2.**
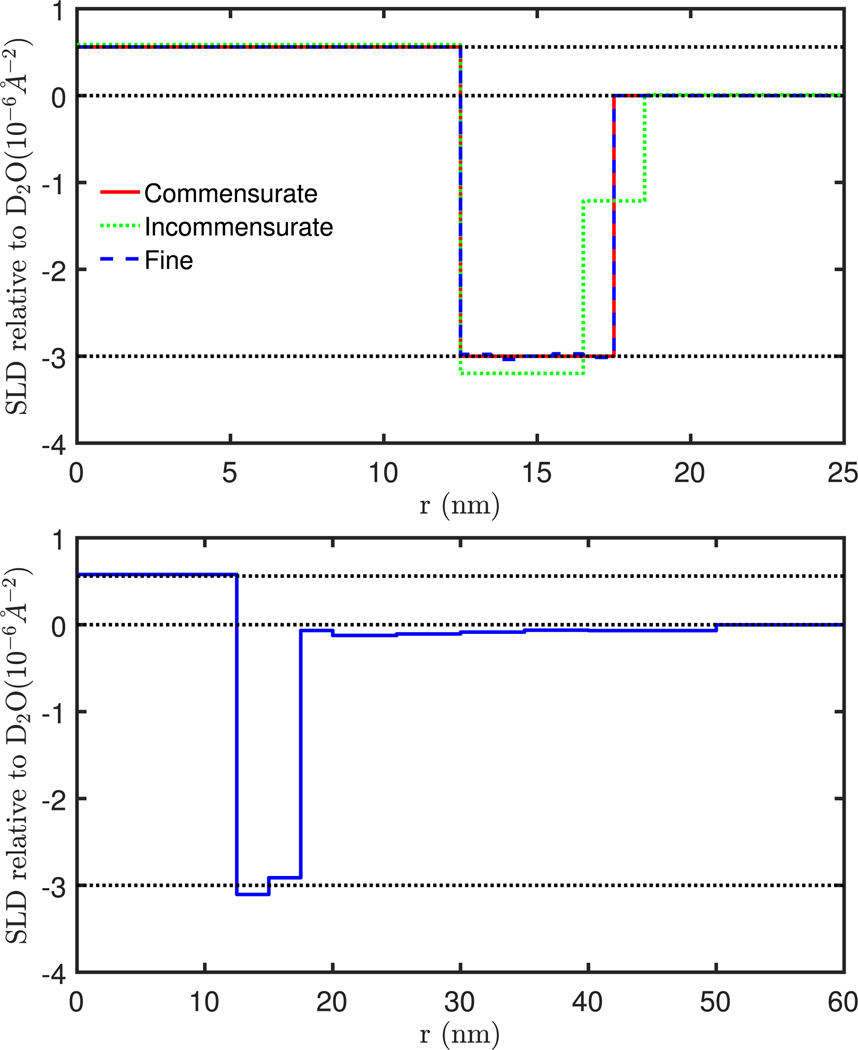
SLD profiles inverted from simulated scattering data for single particles (top) and dimers (bottom). The horizontal dashed lines represent the input SLDs of the core (0.56 · 10^−6^ Å^−2^), shell (−3.0 · 10^−6^ Å^−2^), and solvent (0).

To test the limits of the method, inversion of simulated scattering data was also performed. The data was simulated with realistic experimental conditions (Q-range, resolution smearing, statistical noise) and with sample parameters that resemble the expected structure of the core–shell nanoparticles used in the experiment. The results of the simulations are summarized in appendix. The inverted simulated data demonstrates that an SLD profile almost perfectly matching the input values is obtained if the chosen FED corresponds to the sample structure and that even if this is not the case, i.e., if the sample SLD varies within one FED element, the overall structural features are still correctly reproduced (see figure A2 top).

In recent conventional SANS studies of samples similar to the ones studied here [28] it was found that the core–shell particles tended to form two-particle aggregates (dimers). Therefore scattering from dimers was simulated in the same way as the isolated particles. Inversion of the simulated dimer scattering produced unreasonable results unless the inverted spatial range was extended to approximately 60 nm, exactly as for the experimental data. This is a strong indication that dimer formation is happening in the sample and that the correlation from the reference to the structure of the neighboring particle is responsible for the slow decrease in SLD contrast from polymer to solvent in the ≈20–40 nm range. This illustrates that the method is sensitive to even weak correlations and highlights the need for good samples with minimal interparticle interactions.

Inversion of experimental data was performed successfully with four different choices of FED, giving overall consistent SLD profiles for all choices. This shows that the exact details of the FED is not so important, as long as the FED is within the limits of the experimental Q-range and not too coarse to represent the sample structure. The fact that the inversion works with the 2 nm bin size shows that we do obtain a resolution that is better than what could be obtained by conventional SANS by a factor of two.

Nanoscale core–shell particles with a magnetic core and a nonmagnetic shell are relevant for applications in electronics and biomedicine, such as permanent magnets, magnetic hyperthermia and thermally assisted drug delivery. Although the internal structure of such systems is important for their intended function it is difficult to determine the internal structure with conventional methods [29–33]. Our study shows that it is possible to successfully obtain phase sensitive information about the structure of a core–shell system consisting of a magnetic nanoparticle with polymer coating.

The most important difference between PS-SANS and conventional SANS techniques is that; (i) Conventional unpolarized SANS can give information about the orientationally averaged, model dependent shape (e.g., radius of gyration) and chemical composition of a macromolecular object of interest (ii) Polarized SANS can additionally give this information about ferromagnetic components. (iii) PS-SANS can give information about model *independent* shape and composition of a macromolecular object of interest *without* orientationally averaging via the use of a known attached reference structure. Orientationally averaging is of course only relevant for samples without spherical symmetry, and while core–shell structures are of considerable interest, our method can only be considered a modest success until it is used to determine the three dimensional structure of macromolecules in solution. This could potentially be of great importance, especially for proteins that cannot be crystallized and for determining differences between crystallized and in-solution structures, but this cannot be realized using a single spherical reference particle attached to the sample.

To obtain a full three dimensional structure of a molecule one could either use asymmetric reference particles or attach multiple references to the molecule. The references have to be rigidly attached with (nearly) identical position and orientation with respect to the sample structure and it is thus not a simple matter to realize. Therefore the next step could be to use, e.g., rod-shapes particles that do not have spherical symmetry or to tag a molecule with multiple spherical references at known sites.

Finally, we should mention that phase sensitive SANS can be achieved without the combination of magnetic nanoparticles and polarized neutrons. For example, instead of effectively changing the magnetic SLD of the reference by changing the neutron polarization, one could conceivably use a non-magnetic reference, such as a very well determined molecule. If scattering of the compound sample-reference could be measured, as well as the scattering of the sample on its own and of the reference on its own, the sample structure factor amplitude and phase could be retrieved. However, such an experiment would have the disadvantage that the sample may be changed between measurements with and without reference.

## 7. Conclusions

We have successfully performed the first experimental realization of the particular phase-sensitive small-angle neutron scattering method originally described theoretically in [8] using, for this specific test case, a magnetic reference particle in conjunction with polarized beams. Our results demonstrate the feasibility of the method by recovering the structure of an iron-oxide-core polymer-shell particle. We demonstrate that the method is robust and gives structural information with a resolution that is better than expected from a conventional SANS experiment. Our experiments also demonstrate the sensitivity of the method and the importance of the preparation of high quality samples. Our method is directly applicable to magnetic core–shell nanoparticle systems and could be extended to three dimensional structure determination of macromolecules in solution by using anisotropic reference particles.

## Figures and Tables

**Table 1.** Structural- and magnetic particle sizes from FCC paracrystal fits to PASANS data. The given uncertainties represent one standard deviation.

	Radius (nm)	Polydispersity (%)

Nuclear	12.50 (2)	4.6 (2)
Magnetic	11.8 (2)	13.8 (12)

**Table 2.** Expected SLDs for the sample constituents. The SLD for iron oxide (Fe_3_O_4_) is estimated assuming a density of 5.2 gcm^−1^. The SLD of the polymer is expected to be between that of H_2_O and D_2_O.

	$\rho \left(10^{-6}{\text{Å}}^{-2}\right)$	$\rho -\rho_{D_{2}O}\left(10^{-6}{\text{Å}}^{-2}\right)$

D_2_O	6.38	0
H_2_O	−0.56	−6.94
Fe_3_O_4_	6.97	0.59
Polymer	−0.56 to 6.98	−6.94 to 0

**Table 3.** Finite element decompositions (shell binnings) corresponding to the inverted SLDs in figure 7.

	2 nm bins	3 nm bins	4 nm bins	5 nm bins

$R_{1}$ (nm)	12.5	12.5	12.5	12.5
$R_{2}$ (nm)	14.5	15.5	16.5	17.5
$R_{3}$ (nm)	16.5	18.5	20.5	22.5
$R_{4}$ (nm)	18.5	21.5	24.5	27.5
$R_{5}$ (nm)	22.5	24.5	30.0	32.5
$R_{6}$ (nm)	30.0	30.0	40.0	40.0
$R_{7}$ (nm)	40.0	40.0	50.0	50.0
$R_{8}$ (nm)	50.0	50.0	60.0	60.0
$R_{9}$ (nm)	60.0	60.0		

**Figure A1.**
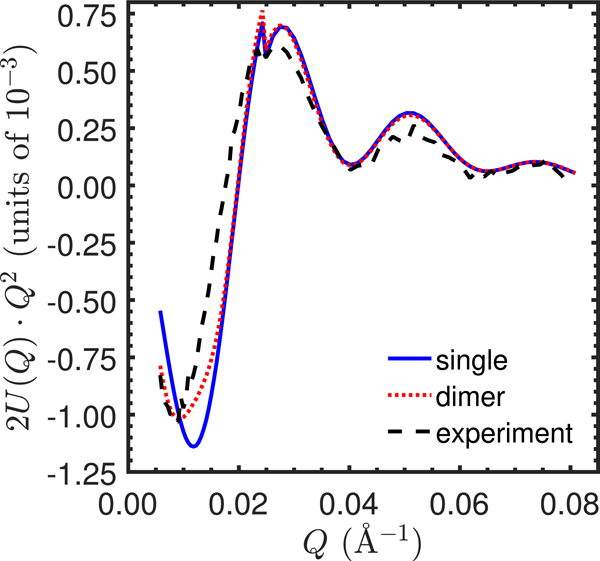
Simulated cross term U(Q) multiplied by $Q^{2}$ for single particles and dimers. The experimental data is displayed for comparison.

**Figure A3.**
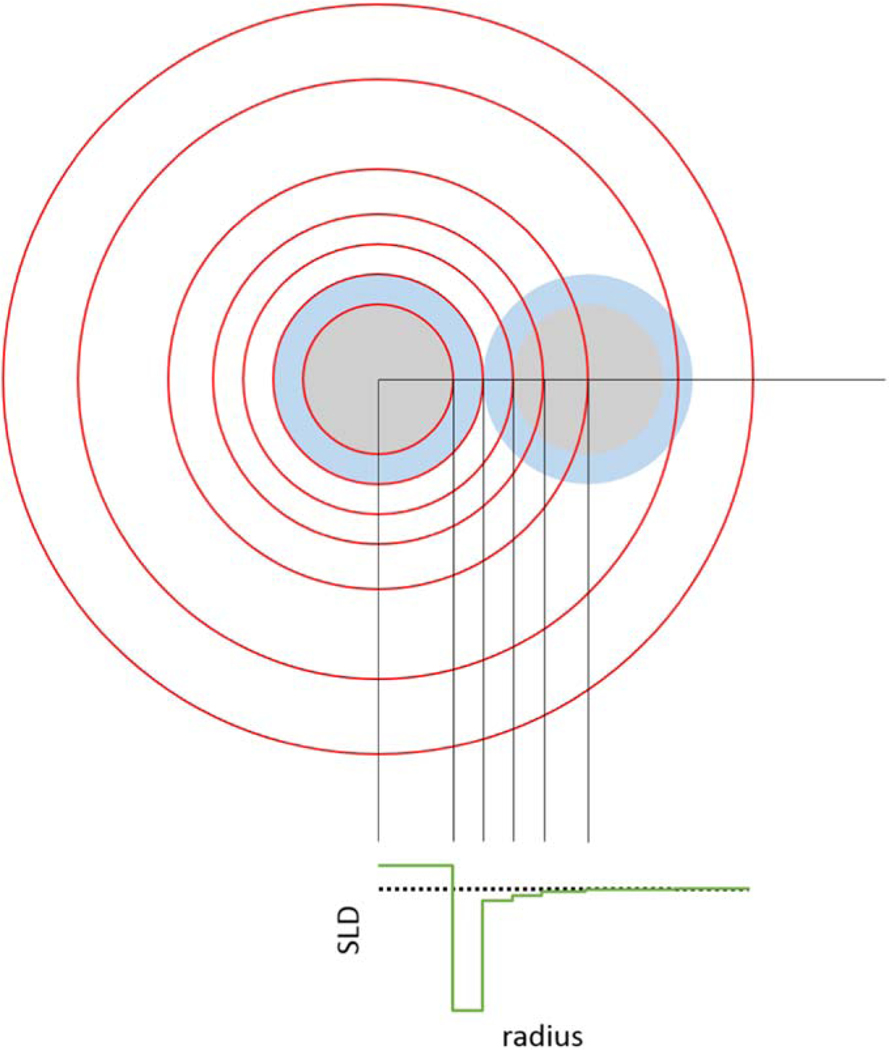
Dimer inversions. From the point of view of the reference, which we imagine as centered in one of the particles in the dimer the SLD in the FED components will be volume averages of solvent SLD and the SLD of the neighboring particle

## Footnotes

7 The identification of any commercial product or trade name does not imply endorsement or recommendation by the National Institute of Standards and Technology.

## Appendix.

### Inversion of simulated scattering data

To test the validity of the results of inverting the experimental data and to study the effect of the choice of FED inversions were also performed on simulated scattering data from a core–shell structure. The simulations were performed using the experimental Q-range and resolution smearing as well as statistical noise. The sample parameters for the simulations were chosen to be comparable to those expected for the sample with a volume fraction of ϕ=0.002, core radius of 12.5 nm, shell thickness of 5 nm, and SLDs of 0.56 · 10^−6^ Å^−2^ and −3.0 · 10^−6^ Å^−2^ relative to the solvent, for the core and shell respectively. The reference was given a radius of 12 nm with 10% polydispersity and a magnetic SLD of 1.46 10^−6^ Å^−2^.

Conventional SANS investigations of samples similar to the ones studied here indicate that the particles may form two-particle aggregates (dimers) in the suspension [28]. To see how this would effect the inversion experiment we simulated both scattering of isolated core–shell particles and dimers of core–shell particles. In the simulations of the dimers the separation between the two particles was equal to one core–shell radius at 35 nm, i.e., the two particles are touching. Orientational averaging was performed for a random average orientation of the dimers.

The simulated difference function U(Q) is plotted in figure A1 for both the single particles and the dimers along with the experimental data for comparison. Interestingly, the shape of the U(Q) experimental curve seems to be better represented by the dimer simulation than by the single particle simulation, indicating that dimer formation might indeed be happening and that it changes U(Q) significantly.

The SLDs were obtained from the simulated U(Q) using equation (23). For the simulated single particle model the inversion was not very sensitive to the choice of binning and a core–shell structure with dimensions and SLDs close to the input parameters was always recovered. In figure A2 (top) the inverted SLD profile for the single particle is shown for three different FEDs. The one labeled ‘commensurate’ has a FED with only three elements that exactly correspond to the simulated structure, i.e., $R_{1}=12.5\;\text{nm}$, $R_{2}=17.5\;\text{nm}$, and $R_{3}=25\;\text{nm}$, where the truncation of the last shell corresponding to the solvent is arbitrary. The FED labeled ‘incommensurate’ has one element corresponding to the core, one entirely inside the shell, one that is partly in the shell and partly in the solvent as well as one entirely in the solvent. Inversion with the commensurate FED exactly reproduces the input SLD. The incommensurate FED results in an SLD of −3.2 · 10^−6^ Å^−2^ for the part that corresponds to the shell (input SLD of −3.0 · 10^−6^ Å^−2^) and −1.29 · 10^−6^ Å^−2^ for the part corresponds partly to shell and partly to solvent, where the expected SLD would be −1.21 · 10^−6^ Å^−2^ (calculated from the volume fraction inside and outside the shell). The inversion labeled ‘fine’ in the figure has an FED with 1 nm thin elements in the shell region and represents the input SLDs extremely well.

When the simulated dimer scattering was inverted using the same FEDs as for the isolated particles the resulting SLDs differed from the input values by more than an order of magnitude. To get the inversion to produce reasonable SLD values the FEDs had to be extended to a radius of approximately 60 nm, exactly as was the case for inversion of the experimental data. The SLD profile from inversion of the simulated dimer scattering (lower panel of figure A1) resembles that of the sample (figure 7) indicating that the nonzero SLD beyond the immediate shell can likely be attributed to correlation to neighboring particles. The sketch in figure A3 illustrates that the SLD distribution of a dimer as seen from one of the particles as reference resembles the results from our inverted experimental data.
